# A New Rat Model of Sacral Cord Injury Producing a Neurogenic Bladder and Its Functional and Mechanistic Studies

**DOI:** 10.3390/biom14091141

**Published:** 2024-09-09

**Authors:** Kaiping Bai, Yanping Hou, Zhiyuan Zhang, Fei Yuan, Xiaoling Huang, Pengtao Liu, Xiangyu Zou, Jie Sun

**Affiliations:** Department of Urology, Shanghai Children’s Medical Center, School of Medicine, Shanghai Jiao Tong University, No. 1678 Dongfang Road, Pudong New Area, Shanghai 200127, China; bkp09024@sjtu.edu.cn (K.B.); houyanping@scmc.com.cn (Y.H.); zhangzhiyuan@scmc.com.cn (Z.Z.); yf13072381838@sjtu.edu.cn (F.Y.); hxl0731@sjtu.edu.cn (X.H.); pengtao_liu@sjtu.edu.cn (P.L.); zouxiangyu@scmc.com.cn (X.Z.)

**Keywords:** neurogenic bladder, sacral spinal cord injury, urodynamics, inflammation, fibrosis

## Abstract

Sacral spinal cord injury (SSCI) can disrupt bladder neuromodulation and impair detrusor function. Current studies provide limited information on the histologic and genetic changes associated with SSCI-related neurogenic lower urinary tract dysfunction (NLUTD), resulting in few treatment options. This study aimed to establish a simple animal model of SSCI to better understand the disease progression. Ninety 8-week-old *Sprague-Dawley* (SD) rats were randomly separated into sham operation and SSCI groups. The SSCI group underwent sacral spinal cord injury, while the sham group did not. Urodynamic and histological assessments were conducted at various intervals (1, 2, 3, 4, and 6 weeks) post-injury to elucidate the disease process. Urodynamic examinations revealed significant bladder dysfunction in the SSCI group compared to the sham group, stabilizing around 3–4 weeks post-injury. Histological examination, including hematoxylin–eosin and Masson’s trichrome staining, correlated these functional changes with bladder microstructural alterations. RNA-seq was performed on bladder tissues from the sham group and SSCI group at 6 weeks to identify differentially expressed genes and pathways. Selected genes were further analyzed using polymerase chain reaction (PCR). The findings indicated a pronounced inflammatory response in the first 2 weeks post-SSCI, progressing to bladder fibrosis at 3–4 weeks. In conclusion, this study presents a reliable, reproducible, and straightforward SSCI model, providing insights into bladder functional and morphological alterations post-SSCI and laying the groundwork for future therapeutic research.

## 1. Introduction

Neurogenic bladder (NB) results from various nervous system disorders and manifests as bladder or urinary tract dysfunction [1]. NB can lead to a range of issues, including altered bladder compliance, bladder wall thickening, vesicoureteral reflux, and potentially renal failure [2]. Clinical symptoms vary depending on the segment of the spinal cord affected by spinal cord injury (SCI). Severe sacral spinal cord injury (SSCI), such as from spina bifida [3], tethered cord syndrome [4], presacral meninges [5,6], and sacral canal cysts [7,8,9,10], can disrupt the micturition reflex arc, resulting in the loss of detrusor reflex, reduced detrusor contractility, and symptoms like urinary retention and difficulty urinating. Prolonged bladder filling can compress blood vessels, reduce blood flow, and cause hypoxia [11]. Research indicates that over half of patients with spina bifida or tethered cord syndrome experience severe urinary tract symptoms [10,12]. Early intervention can improve quality of life and prevent further urinary system damage [10,13]. Additionally, conditions such as sacral cysts, lumbosacral tumors, and complications from spinal surgery can affect the sacral micturition center [14,15,16]. Although sacral spinal injury is relatively common, there is no definitive cure for NB. Clean intermittent catheterization remains the gold standard for managing high urinary tract residue [17]. However, it is burdensome, requiring 4–6 daily insertions, and can lead to complications like urinary tract infections. Current drug treatments offer limited efficacy [18], necessitating further exploration of the disease’s pathophysiological mechanisms and potential treatments.

The bilateral pelvic nerve crush injury (BPNI) model is commonly employed to study neurogenic detrusor underactivity [19,20,21]. This model mimics nerve damage from pelvic surgery by clamping the bilateral pelvic nerves of rats, leading to bladder denervation. However, the BPNI model only addresses spinal nerve root injury and does not adequately represent conditions like meningocele, spinal lipoma, and tethered cord syndrome. Ozsoy O et al. [22] described functional and morphological alterations in neurogenic bladder and motor dysfunction from spinal cord compression. Noritoshi Seysto et al. [23] developed an animal model of bladder inactivity caused by lumbar spinal stenosis, but it requires significant spinal cord exposure, is highly traumatic for the rats, and takes a long time to establish. To address these limitations, we developed a new SSCI model that involves a small back incision and a puncture needle into the intervertebral space (L3–L4). This model more accurately simulates SSCI-induced NB with minimal trauma. We comprehensively evaluated the model’s functional, imaging, morphological, and molecular characteristics to better understand its features and potential for further research.

## 2. Materials and Methods

### 2.1. Animals

All experiments received approval from the Experimental Animal Management and Ethics Committee of the Shanghai Experimental Animal Research Center (Approval No. 202400502). Ninety healthy female *Sprague-Dawley* (SD) rats (8 weeks old, 200~225 g) were acquired from Shanghai Jihui Experimental Animal Breeding Co., Ltd. (Shanghai, China) and housed under standard conditions, with 12 h light/dark cycle, 40% humidity, free access to water and food, and a controlled temperature of 22 ± 2 °C. The rats were randomly assigned to 6 groups of 15 rats each, with groups designated for sham operation or SSCI. Anesthesia for animal operations was achieved using a 3% pentobarbital sodium solution, and isoflurane/ethyl carbamate was used for functional and imaging analyses. Six rats from each group underwent urodynamic analysis. Bladder tissues from six rats per group were collected for morphological and immunohistochemical studies, while the remaining tissues were harvested and stored at −80 °C for subsequent molecular biology experiments. Additionally, bladder tissues from five rats in the sham operation group and the SSCI 6-week group were used for RNA-seq analysis. In accordance with the ARRIVE guidelines (Animals in Research: Reporting In Vivo Experiments), the experimental rats were humanely euthanized by cervical dislocation.

### 2.2. Study Design

The experiment was organized into six groups: sham operation group and post-SSCI at 1 week, 2 weeks, 3 weeks, 4 weeks, and 6 weeks (sham, 1w, 2w, 3w, 4w, 6w). First, we used ultrasound (US) to compare the morphological characteristics of rats in each group. Next, urodynamic evaluations were conducted to assess the micturition patterns in each group. Bladder tissues were collected at their respective time points and analyzed histologically using H&E and Masson’s trichrome staining to evaluate morphological characteristics. Immunofluorescence staining was used to assess the degree of bladder tissue hyperplasia. Ultimately, RNA-seq and bioinformatics analyses were conducted, followed by molecular and histological verification using the bladder tissues from each group.

### 2.3. SSCI Model

**Figure 1:**
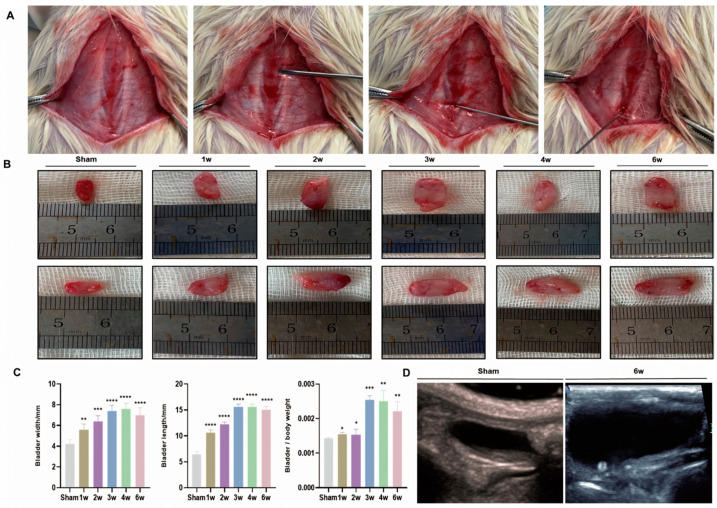
Surgical procedure for sacral spinal cord injury and subsequent bladder shape changes in the SSCI rat model. (**A**) The SSCI surgical procedure. From left to right, the sequence is as follows: The skin is dissected to locate the spinal process; the second image marks the first lumbar vertebra; the third image shows the L3-L4 intervertebral space; and finally, a small needle knife is inserted into this space to damage the spinal cord (fourth picture). (**B**) Comparison of bladder appearance in the sham group and 1 week, 2 weeks, 3 weeks, 4 weeks, and 6 weeks post-operation. (**C**) Weekly comparisons of bladder width, bladder length, and bladder/body weight (g/g). (**D**) Ultrasound comparison of the bladder in the sham group and the SSCI group at 6 weeks post-operation (* *p* < 0.05, ** *p* < 0.01, *** *p* < 0.001, **** *p* < 0.0001 compared to sham rats).

For the surgical procedure, each rat was weighed and anesthetized using 3% pentobarbital sodium solution (2.5 mg/100 g). After shaving the rat’s back, the spine segment was located, and a midline incision was made along the back. Under anesthesia, the third and fourth lumbar vertebral arches were exposed. A small needle knife (0.35 × 25 mm; Yunlong, Wujiang, China) was inserted into the intervertebral space between the third and fourth lumbar vertebrae. Subsequently, a larger needle knife (0.35 × 40 mm; Yunlong, Wujiang, China) was inserted through the same incision to disrupt the spinal cord. The wound was then sutured in two layers with No. 4 silk, and systemic antibiotics (Benzylpenicillin sodium for injection, 100,000 IU/kg, Keda, Fuzhou, China) were administered. Postoperatively, the rats were given a standard diet. SSCI rats received bladder compressions at least twice daily (gently pressing the abdomen with two fingers) to aid urination. In the sham group, the same surgical procedure was performed without inserting the puncture needle. No rats died following the sham and SSCI surgeries (Figure 1A).

### 2.4. Urodynamics

Urodynamic examinations were conducted on each group of rats. All rats were anesthetized using urethane (1 g/kg), the preferred anesthetic for studying urination physiology [24,25]. In brief, a sterile polyethylene catheter (PE-50) was inserted into the bladder dome. Intravesical pressure was measured using this PE-50 catheter, connected to an electrophysiological recorder (BL-420N; TECHMAN, Chengdu, China) with a pressure transducer through a three-way stopcock. An infusion pump (JZB-1800; Fresenius-kabi jianyuan, Changsha, China) was used to pump preheated saline into the bladder at a rate of 0.2 mL/min until saline exuded from the external urinary orifice. Before each infusion, the bladder was gently pressed to expel any residual urine. Leak point pressure (LPP) and maximum cystometric capacity (MCC) were recorded as urodynamic measurement parameters, and bladder compliance was calculated using the formula: bladder compliance = maximum bladder capacity/(LPP-initial intravesical pressure).

### 2.5. Ultrasound

To measure postoperative bladder residue, we used a handheld digital ultrasound imaging system (Hitachi, Japan) to visualize the bladders of rats in the sham group and the SSCI 6W at the end of the postoperative observation period. The hair on the lower abdomen was shaved and removed using hair removal cream. The abdomen was then cleaned using 70% ethanol. Under isoflurane anesthesia (Ryward, Shenzhen, China), the lower abdomen of the rats was scanned to display the bladder’s morphological characteristics.

### 2.6. Immunohistochemistry

Bladders from each group were collected, fixed in 4% formalin, and embedded in paraffin. Paraffin sections (5 μm thick) were deparaffinized in xylene and rehydrated with alcohol. The slides were then stained with hematoxylin–eosin (H&E). Masson’s trichrome staining was performed following the standard procedure. In brief, after dewaxing and rehydration, the tissue sections were immersed overnight in Bouin’s solution or Zenker’s solution and then rinsed with running water. The sections were stained with hematoxylin solution (Harris) or iron hematoxylin for 5–10 min, followed by a brief wash with running water. Differentiation was performed using 0.8–1% hydrochloric acid alcohol, and the sections were washed for several minutes with running water. To enhance blueness, sections were treated with lithium carbonate solution and washed with running water. The sections were then stained with ponceau acid fuchsin solution for 5–10 min and washed with running water. They were treated with phosphomolybdic acid for about 5 min, followed by staining with aniline blue for 5 min without washing. The sections were treated with 1% glacial acetic acid for 1 min and dehydrated with 95% alcohol several times. Finally, the sections were dehydrated with absolute alcohol, cleared with xylene, and mounted with neutral balsam. The bladder smooth muscle content was examined with a microscope (Olympus, Tokyo, Japan), five images were taken randomly, and the bladder smooth muscle content was calculated using Image-Pro Plus (6.0 media Controbernetics, Silver Spring, MD, USA).

### 2.7. Immunofluorescent Staining

Bladder tissue sections were mounted on slides and underwent dewaxing and rehydration before being placed in TBST for antigen retrieval. The sections were then incubated with 10% serum (ANTGene, Wuhan, China; Cat. No: ANT0521) for 30 min at 37 ℃ to block non-specific binding. The primary antibodies (CD68:1:200, Servicebio, Wuhan, China; Cat. No: GB113109; α-SMA:1:200, affinity, Jiangsu, China; Cat. No: AF1032; Collagen I: 1:200, Boster, Wuhan, China; Cat. No: BA0325) were diluted with 10% serum to create the primary antibody working solutions. Each section was incubated overnight at 4 °C with 50–100 µL of the respective primary antibody solution, depending on the tissue size. The following day, the sections were allowed to rewarm at room temperature for 15 min, then rinsed three times with TBST, soaking for 3 min each time. The secondary antibodies (Donkey anti-Rabbit IgG (H+L) Highly Cross-Adsorbed Secondary Antibody, Alexa Fluor™ 594, Thermofisher, Waltham, MA, USA, Cat. No: A21207; Donkey anti-Rabbit IgG (H+L) Highly Cross-Adsorbed Secondary Antibody, Alexa Fluor™ 488, Thermo Fisher, A21206) were diluted with TBST to prepare the working solutions. Each section was incubated with 50–100 µL of the secondary antibody solution at 37 °C for 45 min. After incubation, the sections were rinsed three times with TBST, soaking for 3 min each time. DAPI working solution (1:500; Solarbio, Beijing, China, Cat. No: C0060) was added dropwise (50–100 µL per section, depending on tissue size) and incubated in the dark for 5 min, followed by another rinse with TBST. Finally, the sections were covered with fluorescent mounting medium (Southern Biotech; Birmingham, AL, USA; Cat. No: 0100-01) and stored at 4 °C, protected from light. Microscopic (Olympus, Tokyo, Japan) examination, image acquisition, and analysis were then performed.

### 2.8. RNA-Seq

Five samples each from the sham group and the SSCI 6W were selected for RNA sequencing. RNA extraction was performed using standard extraction methods by Chi-Biotech (Shenzhen, China), followed by rigorous quality control of the RNA samples using quantitative Nanodrop and Agilent 4200 TapeStation to assess RNA integrity. Once the library passed inspection, the preparations were sequenced on a NovaSeq 6000 platform (CHI BIOTECH Co., Ltd., Guangzhou, China), generating 150 bp stand-specific paired-end reads. Before analyzing the expression levels of differential genes, the read counts for each sequenced library were adjusted using the edgeR package through a scaling normalization factor. Differential expression analysis between the two conditions was conducted using the edgeR R package (3.40.2). *p*-values were adjusted with the Benjamini and Hochberg method, setting a corrected *p*-value of 0.05 as the threshold for significant differential expression. Gene Ontology (GO) enrichment analysis of differentially expressed genes was conducted using the clusterProfiler R package (4.6.2), with correction for gene length bias. GO terms (*p* < 0.05) represent significantly enriched. KEGG is a comprehensive database to understand high-level functions and utilities of biological systems at the molecular level, including large-scale molecular datasets from genome sequencing and other high-throughput technologies (http://www.genome.jp/kegg/, accessed on 21 November 2023). The clusterProfiler R package was used to assess the statistical enrichment of differentially expressed genes involved in KEGG pathways.

### 2.9. Real-Time Quantitative Polymerase Chain Reaction (qRT-PCR)

Total RNA was extracted using Trizol reagent (Invitrogen™, Waltham, MA, USA, Cat.No:15596018CN) and then reverse-transcribed into cDNA with the PrimeScript™ RTreagent Kit (Takara Biotech, Kusatsu, Japan, Cat. No: RR037A). Quantitative real-time PCR (qRT-PCR) was conducted using specific primers and PowerUp™ SYBR™ Green Master Mix (Applied Biosystems™, Waltham, MA, USA, Cat. No: A25742) on a C1000 Touch Thermal Cycler equipped with a CFX96 real-time system. Expression data were normalized to GAPDH or β-actin levels, and each qRT-PCR reaction was conducted in triplicate. Primer information is detailed in Table 1.

### 2.10. Protein Extraction and Western Blot

Bladder tissue was homogenized in RIPA lysate (P0013B; Beyotime, Shanghai, China) containing protease inhibitor cocktails (P1005; Beyotime, Shanghai, China). The homogenate was sonicated and then centrifuged. The supernatant was collected, and protein concentration was measured using the BCA assay (P0012S; Beyotime, Shanghai, China). Protein samples (30 µg) were separated on a 10% sodium dodecyl sulfate–polyacrylamide gel and transferred onto polyvinylidene difluoride membranes via an electroblot apparatus. The membranes were blocked for 1 h at room temperature with a 5% non-fat milk solution (Beyotime Biotech, Nantong, China,, Cat. No: P0216). Following blocking, the membranes were incubated overnight at 4 °C with primary antibodies: anti-β-Tubulin (1:1000, Abmart, Shanghai, China, Cat. No: M30109M), anti-IL-1β (1:500, Abcam, Cambridge, UK, Cat. No:ab254360), anti-TNF-a (1:500, Abcam, Cambridge, UK, Cat. No:ab205587), anti-Collagen I (1:100, Proteintech, China, Cat. No: 14695-1-AP), and anti-a-SMA (1:5000, Huabio, Hangzhou, China, Cat. No: ET1607-53). After three washes with tris-buffered saline containing Tween 20 (TBST), the membranes were incubated for 1 h at room temperature with secondary antibodies: Alexa Fluor^®^ 680 AffiniPure Goat Anti-Mouse IgG (H+L) (1:5000; Yeasen; Shanghai, China, Cat. No: 33218ES60) and YSFluorTM 680 Goat Anti-Rabbit lgG (H+L) (1:5000; Yeasen; Shanghai, China, Cat. No: 33118ES60). The membranes underwent three additional washes with TBST before imaging.

### 2.11. Statistical Analysis

Statistical analysis was performed using SPSS 26.0 software. Data are presented as the mean ± one standard deviation (SD), with each experiment conducted at least three times. Statistical analyses for comparisons among more than two groups were performed using either a one-way ANOVA or an unpaired Student’s *t*-test. A two-tailed *p*-value of less than 0.05 was considered statistically significant.

## 3. Results

### 3.1. Changes in Bladder Shape in the SSCI Rat Model

We analyzed the bladder tissue of rats in the sham group and SSCI groups weekly and observed that bladder width increased every week for the first 4 weeks (Figure 1B,C), then decreased in the sixth week. This pattern showed significant growth in the initial 3 weeks, followed by a reduction starting in the fourth week (Figure 1B,C). The bladder weight to body weight ratio slightly increased in the first 3 weeks but did not increase later (Figure 1B,C). This suggests that initial bladder tissue changes were primarily due to edema, whereas collagen accumulation and deposition in the later weeks led to significant weight gain. Meanwhile, rats’ body weight remained stable during the 6 weeks following SSCI. At SSCI week 6, bladder stones were found in all rats with sacral spinal cord injury, likely due to urine retention (Figure 1D).

### 3.2. Urodynamics of the SSCI Rat Model

**Figure 2:**
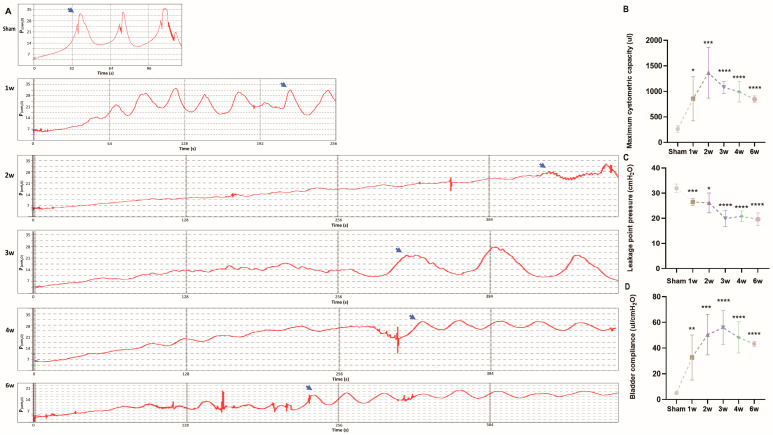
Urodynamics of sham rats and SSCI rats. (**A**) Urodynamic images of the six groups, and arrows indicate leakage of urine. (**B**–**D**) The changes in maximum cystometric capacity, leak point pressure, and bladder compliance in each group (* *p* < 0.05, ** *p* < 0.01, *** *p* < 0.001, **** *p* < 0.0001 relative to sham rats).

To evaluate the impact of the modeling method on the voiding function and weekly changes in micturition dynamics post-modeling, we conducted voiding and micturition dynamics experiments following the protocol outlined by Zheng et al. [26,27]. All six groups underwent successful surgery, with no postoperative fatalities. Urodynamic analysis revealed a rapid increase in maximum cystometric capacity (MCC) within 2 weeks post-modeling, rising from 262 µL ± 69.43 µL to 1361.67 µL ± 496.40 µL. However, MCC at 3, 4, and 6 weeks was lower than that at 2 weeks, gradually decreasing each week, yet remaining larger than the MCC at 6 weeks (842.50 µL ± 66.52 µL vs. sham group; Figure 2A,B, *p* < 0.0001).

Additionally, urodynamic waveform irregularities and the occurrence of autonomous contractions were observed. Following model establishment, leakage point pressure (LPP) decreased from 31.94 cm H_2_O ± 1.67 to 19.57 cm H_2_O ± 2.55. Over the last 3 weeks, LPP tended to stabilize, remaining lower than that of the sham group (Figure 2A,C, *p* < 0.0001). Furthermore, bladder compliance (BC) initially increased post-modeling, with gradual decreases observed after 3 weeks. However, BC remained higher than that of the sham group at 6 weeks (Figure 2A,D, *p* < 0.0001). Urodynamic findings indicated damage to the bladder’s innervating low center following sacral spinal cord injury. In the initial 2 weeks, both detrusor and sphincter activity were impaired, leading to urinary incontinence retention.

### 3.3. Postoperative Pathological Features of SSCI

**Figure 3:**
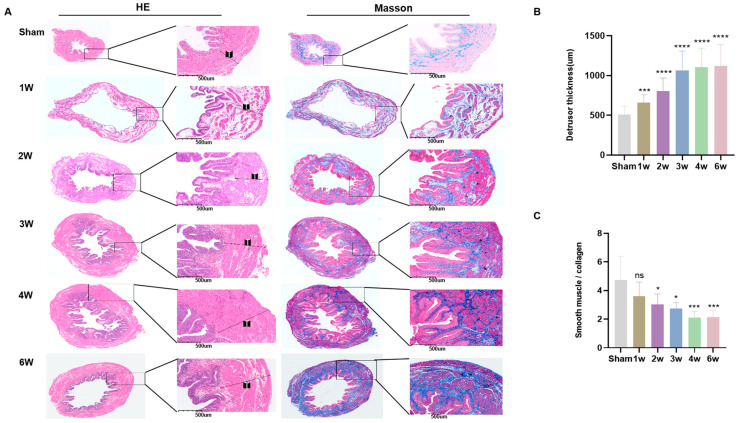
Postoperative pathological features of SSCI. (**A**) H&E staining and Masson staining were conducted in sham rats and SSCI rats. The black arrow denotes extensive fibrosis of the bladder wall, and “T” represents the thickness of the detrusor. (**B**) Weekly comparison of detrusor thickness; (**C**) Weekly comparison of the ratio of smooth muscle to collagen (ns = no significance, * *p* < 0.05, *** *p* < 0.001, **** *p* < 0.0001 compared to sham rats).

Following this, we obtained bladder tissue from each rat group for pathological assessment (Figure 3A). Pathological analysis revealed that within the first 2 weeks post-SSCI, the primary manifestations included subserosal edema and detrusor vacuolization. By the third week after SSCI, observations indicated a reduction in lamina propria and interstitial fibrosis, as well as thickening and hypertrophy of smooth muscle tissue. Furthermore, there was an increase in intramuscular collagen fibers and extensive fibrosis across the entire bladder wall (Figure 3A). Comparative analysis with the sham group revealed significant detrusor muscle thickening and a notable decrease in smooth muscle to collagen post-SSCI. These changes stabilized after 3–4 weeks (Figure 3B,C). Moreover, these alterations correlated with the overall shape and weight of the bladder (Figure 1C).

### 3.4. Postoperative Immunofluorescent Staining of SSCI

**Figure 4:**
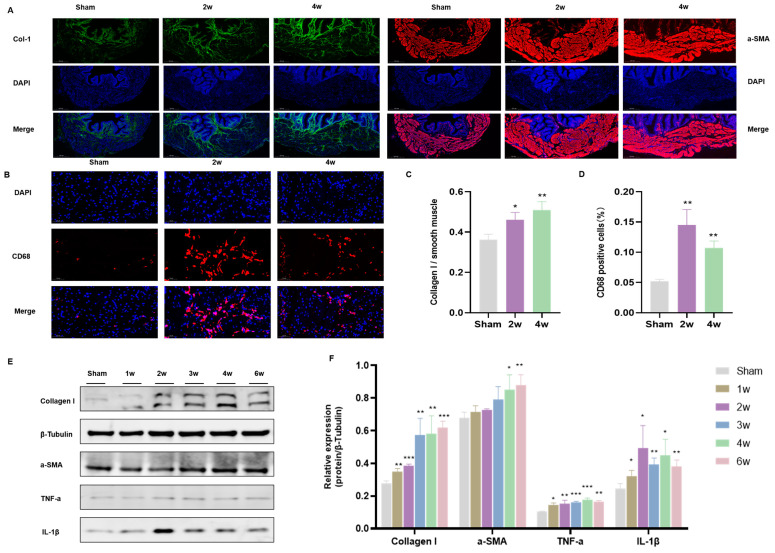
Study of primary pathological processes following SSCI. (**A**) Immunofluorescence staining of col-1 and a-SMA at 2 and 4 weeks post-SSCI. (**B**) Immunofluorescence staining of CD68 at 2 and 4 weeks post-SSCI. (**C**) Comparison of the ratio of collagen 1 to smooth muscle. (**D**) Comparison of CD68 positive cell rate. (**E**) Western blot and (**F**) the relative optical density (* *p* < 0.05, ** *p* < 0.01, *** *p* < 0.001 compared to sham rats). Original images can be found in Figure S1.

Based on the aforementioned findings, we can infer that bladder tissue hyperplasia and inflammatory response were notably active within the initial 2 weeks post-SSCI, gradually stabilizing thereafter, particularly by 3–4 weeks post-injury. Consequently, we selected bladder samples from 2 weeks and 4 weeks post-operation for immunofluorescence staining. Immunofluorescence analysis revealed that collagen I/a-SMA expression at 4 weeks post-operation was higher compared to both 2 weeks post-operation and the sham group (Figure 4A,C). CD68 staining indicated a higher macrophage positive rate at 2 weeks post-operation than at 4 weeks (Figure 4B,D). These results suggest an acute injury phase during the first 2 weeks post-operation, marked by increased macrophage reactivity and subsequent bladder tissue inflammation. Furthermore, we corroborated these findings through Western blot analysis (Figure 4E,F), which demonstrated a consistent trend with the immunofluorescence results.

### 3.5. Transcriptome Analysis Uncovered Different Signaling Pathways in the SSCI Sham Group and the 6-Week Postoperative Group

**Figure 5:**
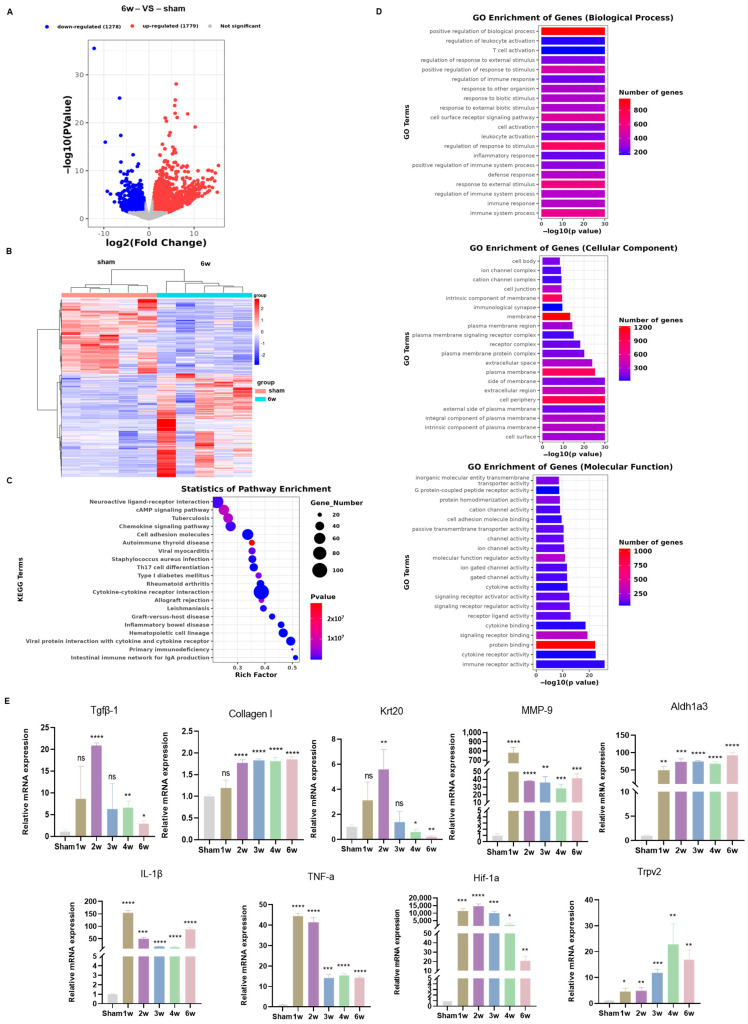
Sham rats and SSCI 6w rats transcriptome analysis and qRT-PCR verification. (**A**) Volcano plot of differential genes between sham rats and SSCI 6w rats; blue denotes decreased expression while red indicates increased expression. (**B**) Heatmap of differential genes in transcriptome data. Blue denotes decreased expression, while red denotes increased expression. (**C**) Bubble chart of enriched Kyoto Encyclopedia of Genes and Genomes (KEGG) analysis of DEGs. (**D**) GO analysis of the sham group vs. post-SSCI 6w rats. (**E**) Nine DEGs validated by qRT-PCR (ns = no significance,* *p* < 0.05, ** *p* < 0.01, *** *p* < 0.001, **** *p* < 0.0001 compared to sham rats).

To delve into the mechanisms underlying the observed urodynamic and morphological changes at various time points post-SSCI, bladder tissues from both the sham operation group and the 6W group were collected for RNA-seq analysis. The heatmap and volcano plot depicting differentially expressed genes (DEGs) are presented in Figure 5A,B, respectively. Comparative analysis with the 6W group post-SSCI revealed 1779 up-regulated genes and 1278 down-regulated genes.

KEGG enrichment analysis of DEGs unveiled significant enrichment in pathways related to cell adhesion, chemokines, cAMP, cytokine–cytokine receptor interaction, calcium signaling, NF-κB signaling pathway, and jak-stat pathway (Figure 5C,D). Subsequently, 9 out of 3057 DEGs were selected for qRT-PCR validation. Their expression patterns mirrored those observed in RNA-seq, affirming the reliability of the RNA-seq data (Figure 5E).

## 4. Discussion

In this research, we developed a novel animal model of the neurogenic bladder using female SD rats, followed by the observation of urinary dysfunction and alterations in bladder morphology. Noteworthy increases in relative bladder weight and shifts in bladder morphology were noted after SSCI, corroborating findings from urodynamic assessments and Masson trichromatic staining. Urodynamic evaluations demonstrated a swift rise in MCC post-SSCI, followed by a gradual decline and stabilization, along with a decrease in LPP and an increase in bladder compliance. Pathologically, SSCI was characterized by heightened collagen deposition, correlating with changes in bladder thickness and wet weight. Previous research suggested a 2-week acute reaction period following spinal cord injury; our pathological and urodynamic findings suggested bladder stability, both functionally and morphologically, after 4 weeks. Subsequently, bladder tissues at 2 and 4 weeks post-SSCI were selected for immunofluorescence staining, revealing a gradual increase in collagen deposition post-SSCI, while macrophage activity peaked at 2 weeks and gradually diminished by 4 weeks. This observation was further validated, indicating more pronounced inflammation at 2 weeks post-SSCI, with gradual recovery by 4 weeks.

Following this, transcriptome sequencing was conducted on bladder tissues from the 6-week post-SSCI and sham groups during a relatively stable state post-injury. Enrichment analysis revealed pathways associated with inflammation and extracellular matrix, findings that were corroborated by qRT-PCR.

SSCI can stem from various factors, including trauma, tethered syndrome, and sacral canal cysts [28,29]. In addition to direct spinal cord injury, other factors may contribute to SSCI and subsequent neurogenic bladder. To comprehensively understand the pathophysiological mechanisms involved, we established a new SSCI model in adult SD rats, incorporating functional, morphological, imaging, and histological evaluations. SD rats are commonly used due to their robust regenerative capacity and strong resistance to infection [30,31]. Given that bladder parameters in normal rats over 8 weeks remain relatively stable, we opted for 8-week-old young SD rats as experimental subjects [32]. Prior methods for establishing neurogenic bladder models, such as the HassanShaker spinal cord transection [33] or Salehi-Pourmehr et al.’s [34] laminectomy at the T9 level, can be laborious. In contrast, our model involves a relatively simple surgical approach, puncturing the L3-L4 intervertebral space to disrupt the spinal cord. This procedure, lasting 10–15 min, boasts minimal mortality rates and requires only twice daily bladder compression to prevent overexpansion and urinary tract infections. Thus, we have developed an effective, user-friendly, reproducible, and reliable animal model of neurogenic bladder with high survivability.

The voiding process relies on the coordinated action of the detrusor and sphincter and is regulated by complex neural control systems located in the peripheral ganglia, spinal cord, and brain [35]. Injury to these regions, whether conical or subconical, can disrupt the integrity of the bladder spinal cord reflex arc, resulting in a flaccid bladder [28] incapable of voluntary urination. Urodynamic testing stands as the gold standard for diagnosing neurogenic bladder disorders [36,37]. Our study revealed a rapid increase in MCC from 1 to 6 weeks post-SSCI, followed by a gradual decline and stabilization, although MCC at 6 weeks remained higher than that in the sham operation group. This phenomenon may be due to the initial elasticity of the bladder after spinal cord injury, followed by a decrease in bladder contractile function and increased tissue fibrosis, leading to a decrease in the MCC. LPP decreased significantly, accompanied by unstable systolic waves, indicating detrusor dyskinesia in SSCI rats. This dyskinesia might arise from detrusor underactivity and weakened micturition contractions due to post-operative neuromodulation disorders. Additionally, bladder compliance surged within the first 2 or 3 weeks, diminishing by 4 weeks and 6 weeks compared to the third week. We speculate that with prolonged observation periods, continued neuromodulation disorders and subsequent contracture development may lead to a further decrease in bladder compliance in SSCI, driven by spasmogenesis, thus potentially exhibiting dynamic changes [10,23,38].

The pathological findings following SSCI indicate that in the early stages of sacral cord injury, neuromodulation disorders lead to subserosal bladder edema and detrusor vacuolization, representing the primary pathological features [21]. Furthermore, we observed a gradual thickening of the bladder wall in the SSCI group, which stabilized after 3 weeks, accompanied by an increase in bladder weight or bladder/body weight ratio. This observation aligns with previous studies, suggesting a compensatory effect of the detrusor muscle [36,38]. The proliferation of rat bladder smooth muscle cells dependent on the PI3K/Akt pathway occurred driven by continuous cardiopulmonary bypass stress [39], while Anderson et al. proposed that detrusor hypertrophy is primarily driven by bladder wall pressure rather than parasympathetic nutrition or spontaneous smooth muscle contractions [40]. This suggests that detrusor hyperplasia may be induced by the increased bladder wall pressure resulting from bladder overfilling due to SSCI. As the disease progresses, stable submucosal collagen secretion becomes apparent at 3 weeks post-SSCI, accompanied by the disappearance of detrusor smooth muscle cell vacuolation, indicative of bladder wall fibrosis, where bladder fibrosis emerges 2–3 weeks later, leading to a gradual decrease in MCC and decreased compliance compared to the preceding 3 weeks.

Based on the results of immunohistochemistry and urodynamic examination, we selected bladder tissues from the SSCI group at 2 weeks and 4 weeks for immunofluorescence staining. The deposition of collagen I gradually increased at both 2 and 4 weeks, consistent with Masson staining results. Similarly, WB tests for Col1 and ⍺-SMA corroborated these findings. CD68 staining revealed a higher macrophage positive rate at 2 weeks post-SSCI compared to 4 weeks, a trend confirmed by WB verification. Notably, the expression of inflammation-related proteins TNF-a and IL-1 β was prominent in the initial 2 weeks post-operation. Prior research has underscored the pivotal role of inflammation in fibrosis progression [41]. In pulmonary fibrosis, the accumulation of extracellular matrix disrupts the balance between collagen synthesis and degradation, exacerbating inflammation [42]. Early injury stages may activate various biosynthesis or biodegradation pathways, initiating excessive extracellular matrix deposition in bladder walls, ultimately culminating in bladder fibrosis and cancer [43,44].

Comparing gene expression profiles of bladders at 6 weeks post-SSCI with those of sham-operated bladders revealed key associations between differential gene expression and signaling pathways. Chemokine, cytokine–cytokine receptor interaction, and cell adhesion pathways were linked to bladder fibrosis post-SSCI. qRT-PCR results indicated early increases in collagen I and significant upregulation of TGF β-1 in the initial 2 weeks post-operation. The TGFβ/Smad signaling pathway has been implicated in chronic bladder outlet obstruction-induced bladder fibrosis [45,46]. High intravesical pressure may further activate protein kinase, modulating cell function changes and promoting fibrosis progression. MMP-9, implicated in tissue fibrosis across various organs, including the heart, liver, and kidneys [47,48,49], promotes fibrosis by inducing EMT [50] primarily via TGF-β1 [51]. Cytokeratin 20 (CK20), also known as keratin 20 (KRT20), expressed in umbrella cells of the normal urinary tract epithelium [52], displayed abnormal increases in the early stages and decreases in the later stages post-SSCI, potentially affecting bladder function. The urothelium of the bladder has a unique response to stretching, facilitating expansion and contraction during filling and emptying, associated with normal voiding [53]. The ALDH family’s activation is crucial for cell survival and protection. It plays an essential role in the conversion of vitamin A to retinoic acid (RA). ALDH is also up-regulated in mammals under oxidative stress and lipid peroxidation [54]. ALDH1A3 expression is linked to apoptosis and angiogenesis [55,56], possibly mediated by the NF-κB and jak–stat pathways. Trpv2, one of the members of the transient receptor potential (TRP) cationic channel family, may influence detrusor excitability and instability and is expressed in the bladder alongside the other four TRP channels (Trpv1, Trpv4, Trpa1, and Trpm8). TRP channels are expressed in the nerve fibers and urothelium of the bladder wall and are sensors for stretching and chemical stimulation [57,58,59]. As the pressure increases, bladder inflammation will occur [60]. The results of qRT-PCR showed that the expression of TNF-a and IL-1 β was consistent with that of WB results, which correlate with NF-κB and immune receptor activity signaling pathways. An increase in the count of cells in the bladder wall in a bladder outlet obstruction model was identified [61], indicating that inflammatory responses may be prominent in early disease stages. Due to nerve damage controlling urination, the bladders of NB patients with NB fill for a long time. For the bladder, its blood perfusion is associated with its filling degree. Overfilled bladder decreases blood flow rapidly, causing ischemia and hypoxia [62], which further severely limit bladder function and activate the downstream genes, including *HIF-1a*, to express [63]. HIF-1a, a transcription factor that is activated under bladder hypoxia, promotes bladder fibrosis.

Limitations persist in this study. Our study focuses on changes in phenotype, gene expression, and pathways during model development, but how these changes occur requires further investigation. It is challenging to clarify the role of specific cell populations in the development of NB induced by SSCI, necessitating further exploration.

## 5. Conclusions

The SSCI rat model is straightforward to produce and possesses the characteristics of a neurogenic bladder, including decreased urination efficiency and lowered detrusor contractile function. We described the clinical starting point for establishing the disease model and comprehensively evaluated the dynamic process of functional, histomorphological, and biomolecular characteristics of detrusor dysfunction associated with sacral spinal cord injury. In this preliminary study, we offer an ideal, quantifiable animal model to characterize detrusor activity associated with SSCI.

## Figures and Tables

**Table 1:** Primer information.

Gene		Primer Sequence (5′ to 3′)	Number of Bases
Krt20	Forward:	TGTCAACGTGGAGGTGGATG	20
	Reverse:	TTCAGAGGACACGACCTTGC	20
Aldh1a3	Forward:	ATGTGAGGTGGAAGAAGGCG	20
	Reverse:	GGCACAATGTTCACCACACC	20
Mmp9	Forward:	CTGAGGCCCCTACAGAGTCT	20
	Reverse:	GTTGTGGAAACTCACACGCC	20
Trpv2	Forward:	ACTACACACGGGGCTTTCAG	20
	Reverse:	CGCTCATCAGGGGTACCATC	20
Tgfβ-1	Forward:	GACTCTCCACCTGCAAGACC	20
	Reverse:	AGCCCTGTATTCCGTCTCCT	20
Hif-1a	Forward:	CGATGCCCTGACTCTGCTAG	20
	Reverse:	GGAGGGCTTGGAGAATTGCT	20
IL-1β	Forward:	CTCACAGCAGCATCTCGACAAGAG	24
	Reverse:	CACACTAGCAGGTCGTCATCATCC	24
TNF-α	Forward:	GCATGATCCGAGATGTGGAACTGG	24
	Reverse:	CGCCACGAGCAGGAATGAGAAG	22
Collagen I	Forward:	CACTGCAAGAACAGCGTAGC	20
	Reverse:	AAGTTCCGGTGTGACTCGTG	20
GAPDH	Forward:	TGACTCTACCCACGGCAAGTTCAA	24
	Reverse:	ACGACATACTCAGCACCAGCATCA	24

## Data Availability

Data in this study can be obtained by request and with permission from the corresponding author.

## Supplementary Materials

The following supporting information can be downloaded at: https://www.mdpi.com/article/10.3390/biom14091141/s1, Table S1: DEGs of the sham group vs. SSCI 6w; Table S2: GO/KEGG analysis of the sham group vs. SSCI 6w; Figure S1: Original Western blots for Figure 4E.
