# Supplementary figures and images for: A New Rat Model of Sacral Cord Injury Producing a Neurogenic Bladder and Its Functional and Mechanistic Studies
Source: Biomolecules. 2024 Sep 9;14(9):1141. doi: 10.3390/biom14091141 (PMC11429646; doi:10.3390/biom14091141)

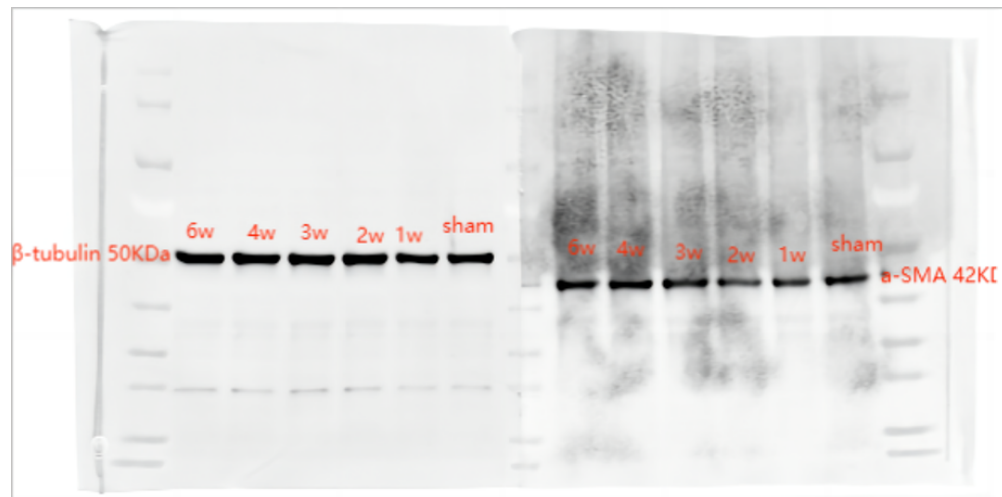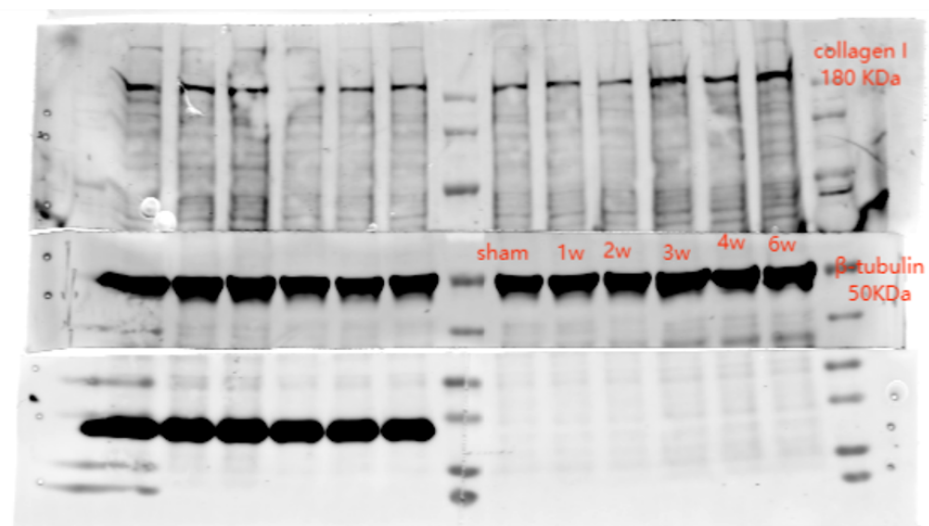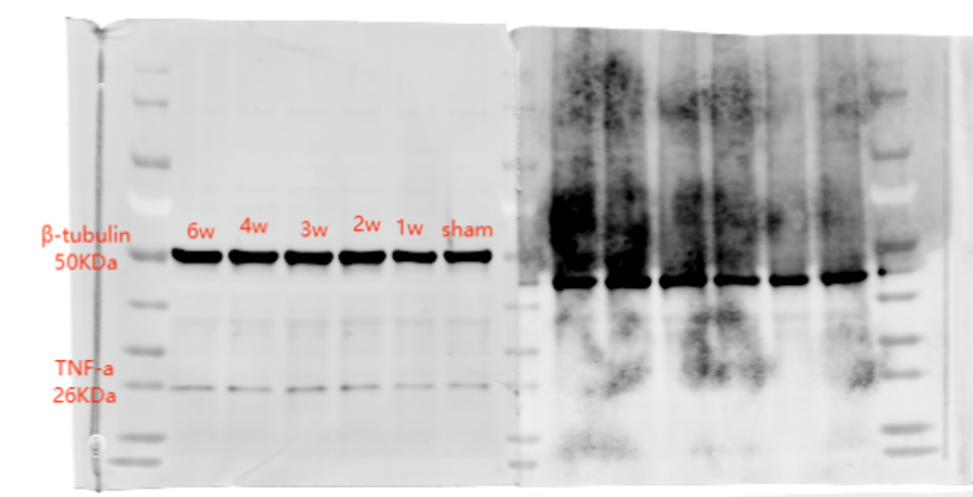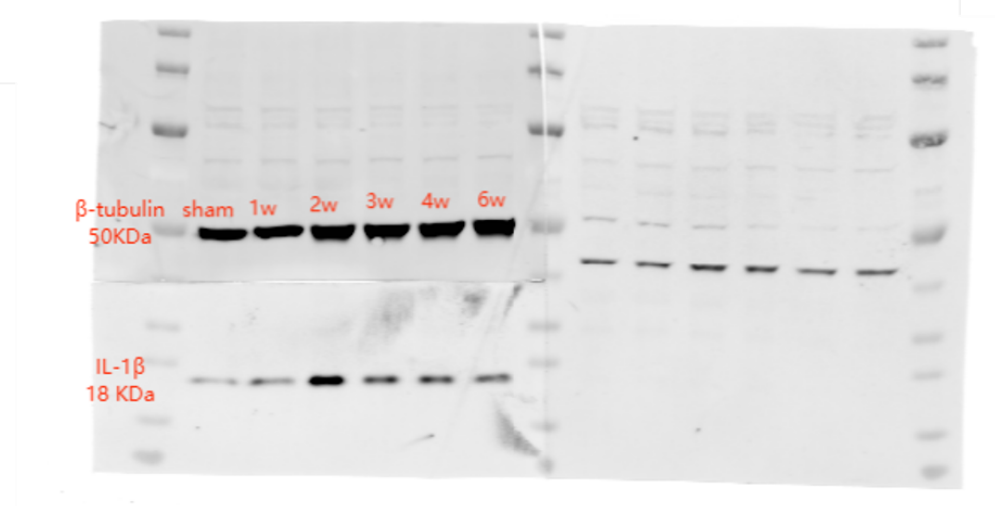

Supplement: Supplementary file 1 [file biomolecules-14-01141-s001.zip › biomolecules-3150369-Figure S1 original-images.pdf]
